# Effects of Two Prey Species Combinations on Larval Development of the Predatory Ladybird *Cheilomenes propinqua*

**DOI:** 10.3390/insects15070484

**Published:** 2024-06-28

**Authors:** Andrey N. Ovchinnikov, Antonina A. Ovchinnikova, Sergey Y. Reznik, Natalia A. Belyakova

**Affiliations:** 1 Zoological Institute, Russian Academy of Sciences, Universitetskaya 1, 199034 St. Petersburg, Russia; anovchi@gmail.com (A.N.O.); antoninaovch@gmail.com (A.A.O.); 2 All-Russia Institute of Plant Protection, Russian Academy of Sciences, Podbelskogo 3, 196608 St. Petersburg, Russia; belyakovana@yandex.ru

**Keywords:** biological control, mass rearing, feeding, mixed diets, changing diets, larval development, adult size, *Cheilomenes propinqua*, *Myzus persicae*, *Sitotroga cerealella*

## Abstract

**Simple Summary:**

The selection of an appropriate diet is a key component of methods for the mass rearing of biocontrol agents. It is known that feeding on mixed, alternating, or changing foods often favor insect development. We investigated the effects of various combinations of high-quality (the green peach aphid) and low-quality (eggs of the grain moth) foods on the development of a predatory ladybird *Cheilomenes propinqua.* The benefits of mixed foods to larval development were only found in some treatments with limited supplies of aphids. Feeding on various combined diets never resulted in higher survival, faster development, or a larger size of emerging adults than those observed for feeding on unlimited amounts of aphids. We conclude that if necessary (for example, in the case of temporary shortage or a lack of aphids in mass rearing facilities or in the case of preventing release of adults for biological control of pests in greenhouses), *C. propinqua* larvae can be fed with grain moth eggs by replacing, mixing, or alternating with aphids, although this will result in a decrease in pre-adult survival, rate of development, and the size of emerging adults. On the other hand, even a minimal addition of aphids can have a substantial positive effect on larvae fed with grain moth eggs.

**Abstract:**

Feeding on mixed, alternating, or changing diets often favor insect development. With the aim to optimize mass rearing and use for the biological control of insect pests, we investigated the effects of various combinations of high-quality (the green peach aphid *Myzus persicae*) and low-quality (eggs of the grain moth *Sitotroga cerealella*) foods on the larval development of a predatory ladybird *Cheilomenes propinqua.* In the first experiment, eggs and aphids were mixed in different proportions; in the second experiment, larvae switched from feeding on aphids to feeding on eggs. Although the beneficial additive effect of mixed foods was detected in some treatments with limited diets, feeding on various combinations of eggs with aphids never resulted in higher survival, faster development, or a larger size of emerging adults than those observed for feeding on unlimited amounts of aphids. For the practice of biological control, we conclude that, if necessary (for example, in the case of temporary shortage or a lack of aphids in mass rearing facilities or in the case of preventing release of *C. propinqua* adults in greenhouses), *C. propinqua* larvae can be fed with grain moth eggs by replacing, mixing, or alternating with aphids, although this will inevitably result in a proportional decrease in pre-adult survival, rate of development, weight, and size of the emerging adults. On the other hand, even a minimal addition of aphids can have a substantial positive effect on larvae fed with grain moth eggs.

## 1. Introduction

The selection of an appropriate diet is a key component of methods for the mass and laboratory rearing of biocontrol agents [1,2,3,4,5]. From an economic point of view, this selection is often a special case of a general ‘price–quality’ schema [6], that is, a choice between highly preferred and most suitable but more expensive natural foods, and relatively cheap but usually much less preferred and less suitable factitious diets. In such situations, many authors have investigated the potential of mixed diets including two or several foods of different qualities [7,8,9,10,11,12,13,14,15,16,17,18,19,20,21,22,23,24,25,26,27]. Much more rarely have alternating [27,28] and changing (switching) [16,29,30,31] diets been tested. Often [7,8,9,10,11,13,14,16,20,22,24,27], although far from always [15,17,18,19,23,25,28], a substantial positive additive effect was observed in these studies: feeding on mixed, alternating, or changing diets resulted in better survival, faster development and maturation, larger individuals, higher fecundity, etc. than feeding on each of their components.

Polyphagous predatory ladybirds are widely used for the biological control of insect pests particularly because of their broad food specificity: one and the same agent can be used against various pests [1,2,3,4,32,33,34]. Indeed, both in laboratory and under natural conditions, many ladybird species can feed on very different animal and plant substances [4,34,35,36,37,38]. However, although different foods can be consumed, their relative suitability can vary drastically. Hodek [35,36] suggested that all acceptable foods of predatory coccinellids could be roughly divided into (1) ‘essential’ foods that ensure the successful completion of larval growth and the development, maturation, mating, normal oogenesis and oviposition by females, and (2) ‘alternative’ foods that can be used merely as a source of energy for the long-term survival of adults. Under natural conditions, the essential prey of different coccinellid predators are most often aphids, soft scales, thrips, mites, and other foods rich in proteins whereas alternative foods are floral nectars, fruit juices, and other plant substances high in carbohydrates. Moreover, essential foods can differ markedly in their preference and suitability for larval development and the reproduction of adult ladybirds [4,8,20,35,36,38,39,40,41,42,43]. As above-mentioned, the laboratory and mass rearing of the natural prey of ladybirds is usually a costly process because of the necessity of growing their host plants. Therefore, factitious (not natural) essential foods, in particular, the eggs of easily reared lepidopteran pests of stored products such as the Mediterranean flour moth, *Ephestia kuehniella* Zeller, and the Angoumois grain moth, *Sitotroga cerealella* (Olivier), are often used [8,10,20,35,36,38,39,40,42,43,44].

Over the past few years, we have studied a predatory ladybird *Cheilomenes propinqua vicina* Mulsant (=*Cydonia (Cheilomenes) vicina* Mulsant) that is widely distributed in subtropical and tropical regions of Africa and Southwest Asia [45]. This polyphagous predator, often reaching high population densities, is rightly considered by many authors as an important natural enemy of various agricultural insect pests. In particular, it was recorded as a predator of *Aphis gossypii* Glover on pear trees in Egypt [46], on guava trees in South Africa [47], eggplants in India [48]; citrus psylla *Trioza erytreae* (Del Guercio) in South Africa [49]; mealybugs on guava trees in Egypt [50]; the cotton mealybug *Phenacoccus solenopsis* Tinsley on various agricultural plants in Israel [51]; mealybugs and green scales on coffee plants in Tanzania [52]; Coccidae infesting fruit trees in Egypt [53]; whiteflies on cassava in Kenya [54]; various insect pests of grapevine [55]; and aphids, mealybugs, and soft scales on various cultural plants in Egypt [56,57,58], Sudan [59], Benin [60], and India [48]. Such a broad range of prey including a number of important pests of protected crops, wide habitat specificity, and large geographic range allow the hope that *C. propinqua* can be successfully used as an effective agent for biological control in greenhouses. In addition, *C. propinqua* larvae and adults show a rather high voracity: when feeding on *A. gossypii*, the larvae of this relatively small (adult body length 4–5 mm) ladybird consumed a total of about 500 aphids during their development whereas the females consumed 40–80 aphids per day with a total of about 2000 individuals per female [43,61,62,63]. Indeed, pilot trials showed that *C. propinqua* is able to successfully control *Aphis fabae* Scop on kalanchoe crops in greenhouse conditions [61].

Moreover, preliminary biological studies have shown that this predatory ladybird combines rapid development (10–11 days from the egg to adult stage under the optimal conditions) with high fecundity (15–30 eggs per day with a total fecundity of about 1000 eggs per female), suggesting a high potential for mass rearing [43,61,62,63]. However, the development of optimal methods for the mass rearing and application of a biocontrol agent requires basic information on its biological peculiarities. Earlier studies concerned the influence of temperature, aphid prey species, and its host plant on the rate of *C. propinqua* pre-adult development, adult size and longevity, female maturation, and fecundity [57,64]. Influences of photoperiod, temperature, and diet on the induction of reproductive (adult) diapause [43], the transportation and storage potential of females [65], and on the prey search activity of adults [66] have also been investigated. A special study was devoted to the comparison of the pre-adult mortality, rate of development, fecundity, net reproduction rate, rate of increase, and other biological parameters in individuals fed on different prey species [59]. In these studies, *C. propinqua* larvae and adults were fed either by preferred high-quality essential prey species such as aphids *Aphis fabae* Scop. [63], *A. gossypii* Glover [57,59], *A. nerii* Boyer de Fonscolomb [59,64], *Melanaphis sacchari* Zehntner [59], and *Myzus persicae* (Sulzer) [43,65,66] or by also essential but much less preferred and less suitable low-quality food such as the eggs of the grain moth *S. cerealella* [43,65,66]. The effects of mixed, alternating, or changing diets on *C. propinqua* larvae and adults have not been investigated, although these approaches have been successfully used with many other mass-reared insects from different orders [7,8,9,10,11,12,13,14,15,16,17,18,19,20,21,22,23,24,25,26,27,28,29,30,31].

It should be also mentioned that mixed diets can be interesting to biocontrol practitioners in two quite different aspects. First, as noted above, feeding on mixtures of different foods can be a promising method for the laboratory and mass rearing of *C. propinqua*. Second, it is expected that *C. propinqua* could be used for the biological control of aphids in greenhouses by the so-called ‘standing army’ method, that is, preventing release of adults not after but before the substantial increase in pest population density [33,67,68,69,70,71,72,73,74,75]. This approach is currently considered very promising, but its application usually requires the regular supplementation of factitious food to promote the survival of the released biocontrol agents at low pest population density or even in the absence of pests (potential prey). In the case of *C. propinqua*, grain moth eggs could be used for this supplementation, whereby the ladybirds would feed on the eggs with the possible addition of some quantity of aphids or other high-quality prey. Therefore, we have quite recently studied the effects of mixed diets (combinations of eggs of the grain moth *S. cerealella* and the green peach aphid *M. persicae* in different proportions) on the reproductive maturation and fecundity of *C. propinqua* females. Mixed diets of this type (low-quality food with the addition of different proportions of high-quality prey) have been used in a number of earlier studies [20,76,77]. Our previous experiments revealed that some of the tested mixed diets can be considered as promising foods for *C. propinqua* females, although the economic feasibility of this method is not clear yet [78]. With regard to basic entomology, the authors of recent reviews on the ecophysiology of predatory ladybirds noted that the additive effects of mixed diets were found only in some of the studies on this subject, and the reasons of this variability are also still not clear [4,18,23,34,35,37]. Therefore, any new data on this topic can be used for further meta-analysis.

The aim of the present study was to evaluate the suitability of mixed and changing diets consisting of different proportions of green peach aphids and grain moth eggs for *C. propinqua* pre-adult development and the influence of these diets on the biological parameters of the emerged adults. The working hypothesis of our study was that mixed food will be the optimal diet for the development of *C. propinqua*.

## 2. Materials and Methods

### 2.1. Insects

The experiments were performed in 2023–2024 with a laboratory population of *C. propinqua* that originated from 42 adults collected in September 2015 in Alexandria, Egypt (31.200391° N, 29.9155046° E) and then reared at the Biocontrol Laboratory, All-Russian Institute of Plant Protection on the wheat aphid (*Schizaphis graminum* Rond.) at a temperature of about 24 °C and the photoperiod of L:D = 16:8 (hereafter, light and dark periods in h are given). Before the experiments, the ladybirds were reared for about three years at the Laboratory of Experimental Entomology, Zoological Institute RAS at a temperature of 25 °C and L:D = 16:8 on the green peach aphid *M. persicae*, which were reared on *Vicia faba* L. seedlings.

### 2.2. Experimental Design

#### 2.2.1. General Methods

At the start of each experiment, a group of about 100 larvae hatched over 24 h from eggs laid by 40–50 *C. propinqua* females of the main laboratory population was reared at 25 °C and L:D = 16:8 on the green peach aphid in transparent plastic containers (27 × 38 × 27 cm) covered with cotton cloth. After emergence, adults were sexed using morphological differences of the terminal sternites: males have a straight margin of the last abdomen sternite while females have a convex distal margin [79,80,81]. In addition, females have a distinct black area along the anteromedian margin of the head [82]. Then, the adults were kept in Petri dishes (90 × 15 mm) at the same temperature and day length in groups of 6–10 individuals (equal number of males and females) and fed one of the two diets: (1) larvae and adults of the green peach aphid *M. persicae* on a *V. faba* seedling (hereafter ‘aphids’) and (2) eggs of the Angoumois grain moth *S. cerealella* glued by 30% sugar solution to a piece of hard paper and an Eppendorf tube filled with water and plugged with a cotton ball (hereafter ‘eggs’). Eggs laid by females fed on each of the two diets were collected daily within 2–4 h after light-on and incubated separately in Petri dishes of the same size under the same conditions. Hatched larvae of the first instar were kept in the same Petri dishes until molt to the second instar. Larvae of the first instar were not fed; the survived individuals cannibalize eggs and other larvae, which is normal for predatory Coccinellidae [35,36,37,38]. Larvae of the second instar were collected daily within 2–4 h after light-on, then reared individually in Petri dishes that were the same size at the same photothermal conditions, and fed on different diets according to the experimental design (see Section 2.2.2 and Section 2.2.3). All other conditions in different treatments of each experiment were exactly the same.

**Figure 1:**
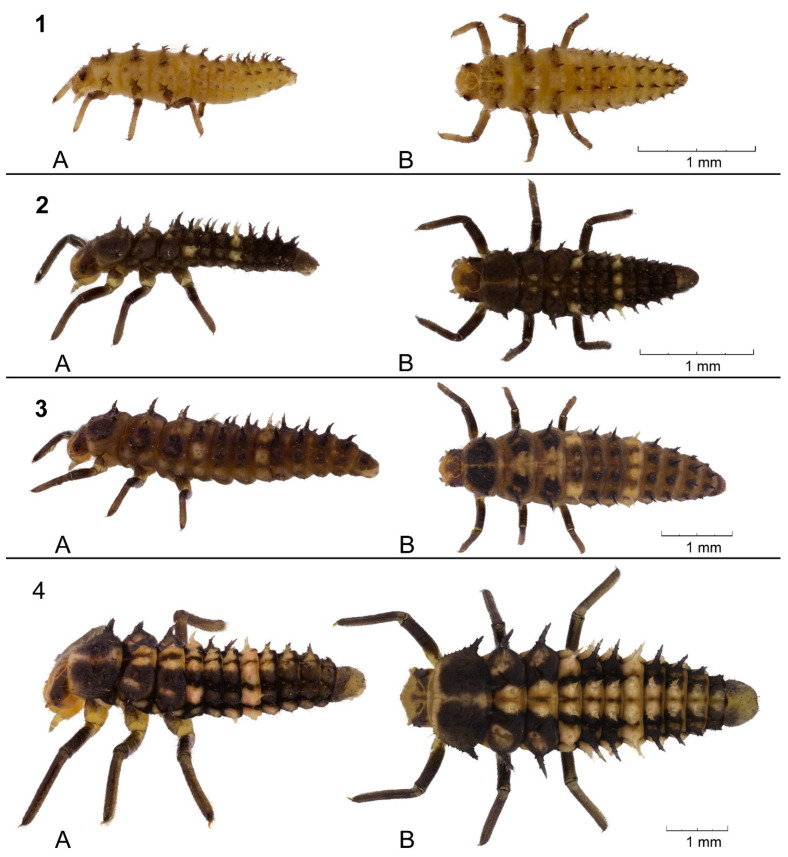
*Cheilomenes propinqua* larvae of different instars. 1—first instar larva, 2—second instar larva, 3—third instar larva, 4—fourth instar larva. (**A**)—lateral view, (**B**)—dorsal view. Photos by A.N. Ovchinnikov.

Each larva was checked daily (2–4 h after light-on) for survival and instar change. Instar change was determined by the presence of shed exuvium and by changes in the color, size, shape, and positions of various larval elements of armature on the dorsal and lateral sides of the thoracic and abdominal segments such as the scoli and parascoli (Figure 1). It is known that most coccinellids have four larval stages, and in the first instar larvae, scoli are shorter, having a small number of branches and longer setae [83,84]. The parascolus is shortened, and there is a modified scolus, (a type of branched projection of the body), the length of which is usually no more than three times its width. Each branch of the scolus bears one thick seta at the distal end. Short descriptions of the larval instars (see also Figure 1) are provided, and the terminology of the different structures on the body of larvae follows Gage’s nomenclature [85].

- First instar larva. Body is elongated, cylindrical, and tapered, with whitish yellow integument. Larval elements of armature are weakly sclerotized. Parascoli are short (about 0.06 mm) and thick.
- Second instar larva. Body and armature of the body surface are sclerotized, pigmented with whitish yellow areas of body. Parascoli are about 0.14 mm, well sclerotized, darker than those on the first instar.
- Third instar larva. Larvae are similar to the second instar in structure and color, body shape is cylindrical and slightly elongated; parascoli are about 0.2 mm, of the same color as the integument.
- Fourth instar larva. Tegument is dark brown with well-demarcated whitish yellow areas of body. Parascoli are brighter colored, larger (0.3–0.4 mm), and more sclerotized than those of the third instar.

Pupation and adult emergence were also recorded every day at the same time (2–4 h after light-on). Freshly emerged adults were immediately weighted with an analytical electronic balance (OHAUS PA-64C) with an accuracy of 0.1 mg and sexed. Then, the maximum body width (with closed elytra) was determined using an MSP-2 stereomicroscope with an accuracy of 0.05 mm as an estimation of adult size. Thus, for each experimental treatment (that is for each tested diet), the survival and mean time of development from the second larval instar to the adult stage and the mean size and weight of emerging adults were determined. Considering that size and weight are closely correlated with the fecundity and voracity of predatory ladybirds [4,14,41,86,87,88,89], we can conclude that the combination of the parameters recorded in our study can be used as a proxy for both the potential of intensive mass rearing and the efficiency of application for the biological control of insect pests.

#### 2.2.2. The First Experiment (Mixed Diets)

In the first ‘mixed diets’ experiment, each larva was fed on the same diet throughout its life. In different treatments, different mixed and pure diets were used. All larval diets consisted of one or both of the two prey species: ‘aphids’ (larvae and/or adults of the green peach aphid *M. persicae*) and ‘eggs’ (eggs of the Angoumois grain moth *S. cerealella*). Eggs (if any) were always provided in excess, whereas aphids in certain diets were limited. This can be explained by the fact that in mass rearing, eggs are considered as a relatively cheap, low-quality factitious diet in contrast to aphids, which are a high-quality but much more expensive food source [78]. Regarding preventing colonization, in greenhouses, eggs can be regularly supplemented in sufficient quantity, whereas the population density of aphids (pests) is expected to be unpredictable and usually low, if not zero. Therefore, the different diets included four levels of aphid content: zero, low (5–10% of the average daily demand for a given day of larval development), medium (about 50% of the average daily demand), and high (unlimited, or more exactly substantially higher than the average daily demand for a given day of development). It should be noted that when planning our experiments, we did not use the physiological (larval instars) but absolute (days) time scale. The reason for this is that the physiological time scale, although more natural, would be too complicated for a practical application. Therefore, it should be considered that our data are valid for the temperature of 25 °C, whereas lower or higher temperatures would require a corresponding elongation or shortening of the time scale. Thus, based on the average daily demands determined in our (unpublished) pilot tests, the quantities of aphids and eggs included in the mixed diets were determined (Table 1). The grain moth eggs were glued by a 30% sugar solution to a piece of hard paper (10 × 15 mm); the amounts of grain moth eggs and sugar solution were kept constant for all treatments in the experiment. Larvae fed on all diets that included eggs were offered an Eppendorf tube filled with water and plugged with a cotton ball. Aphids (if any) were provided on a *V. faba* seedling (10–30 mm in length); if aphids were not included in the diet, an empty (not infested by aphids) *V. faba* seedling was placed in a Petri dish. In combination, two diets of maternal females and seven larval diets led to 14 treatments in the first experiment. Each treatment was performed on 52–58 larvae with a total of 775 individuals, 532 of which (41–51 per treatment, with the exception of the LA diet) developed to the adult stage.

#### 2.2.3. The Second Experiment (Changing Diets)

In the second ‘changing diets’ experiment, all larvae were fed on pure (unmixed) diets (either eggs or aphids) and both foods were always provided in excess. However, the diet was not constant throughout the period of development: initially, the larvae fed on aphids and then switched to feeding on eggs. The difference between the experimental treatments was in the duration of the period of feeding on aphids: 1, 2, 3, 4, or 5 days. In addition, two controls were performed: (1) larvae that constantly fed on eggs (the duration of the period of feeding on aphids was zero) and (2) larvae that constantly fed on aphids (the duration of the period of feeding on aphids was more than 5 days). As shown by our (unpublished) pilot experiments and confirmed by the present study, *C. propinqua* larval development from the second instar larva to the pupal stage at 25 °C lasted for 7–10 days, depending on diet. In particular, in larvae constantly fed on aphids, this period constituted about 7 days. As in the first experiment, aphids were provided on a *V. faba* seedling (10–30 mm in length), and the grain moth eggs were glued by a 30% sugar solution to a piece of hard paper (10 × 15 mm). All larvae fed on eggs were also offered an Eppendorf tube filled with water and plugged with a cotton ball and an empty (not infested by aphids) *V. faba* seedling. In combination, two diets of maternal females and seven larval diets (five experimental treatments and two controls, see Table 2) led to 14 treatments. Each treatment of the second experiment was performed on 56–67 larvae with a total of 846 individuals, 668 of which (42–54 per treatment) reached the adult stage.

### 2.3. Statistical Analysis

Statistical analysis was performed with SYSTAT 10.2 (Systat Software Inc., Richmond, VA, USA). Non-parametric data were analyzed with the chi-square test (when necessary, the Mantel–Haenszel adjustment was used). Parametric data were not normally distributed and were therefore ranked before ANOVA. Single pairwise comparisons of the parametric data were made with the Kruskal–Wallis test; multiple pairwise comparisons with Tukey’s HSD test on ranked data. Other details of statistical analysis are given in the figures and tables.

## 3. Results

### 3.1. The First Experiment (Mixed Diets)

#### 3.1.1. Pre-Adult Survival and Sex

**Figure 2:**
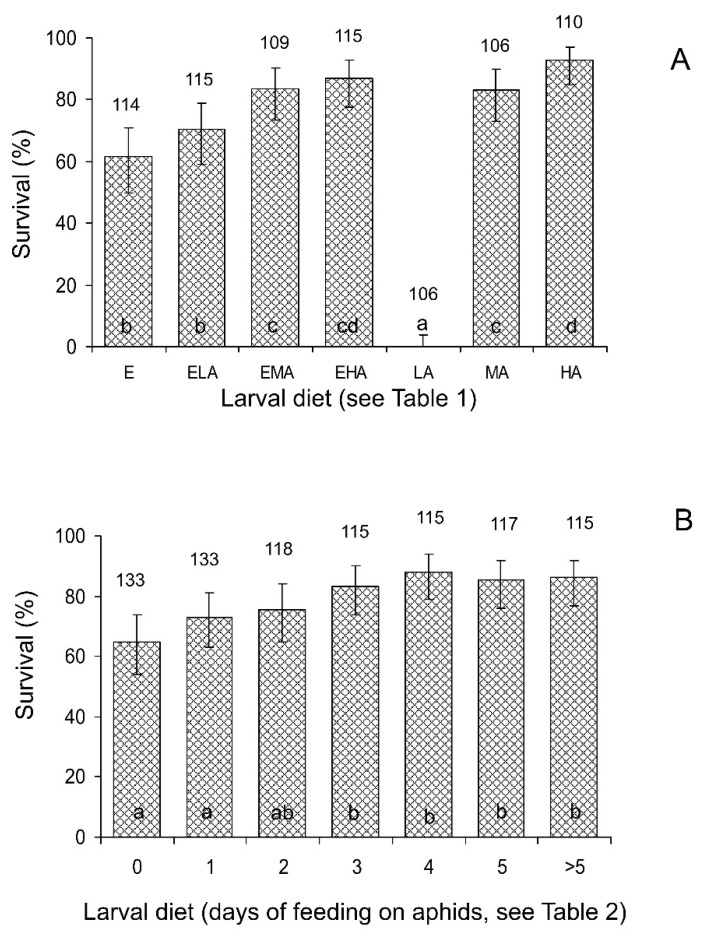
Influence of diet on the survival of *Cheilomenes propinqua* larvae from the second instar to the adult stage. Diets are indicated below the x-axis. (**A**) The first (mixed diets) experiment; abbreviations of diets: E—eggs of the grain moth, ELA—eggs of the grain moth and low quantity of aphids, EMA—eggs of the grain moth and medium quantity of aphids, EHA—eggs of the grain moth and high quantity of aphids, LA—low quantity of aphids, MA—medium quantity of aphids, HA—high quantity of aphids (see Table 1 for more detailed description of diets). (**B**) The second (changing diets) experiment; the numbers below the x-axis indicate the number of days when the larvae fed on aphids before the switch to feeding on eggs (see Table 2 for a more detailed description of diets). Data shown are percents and 95% confidence intervals. Sample sizes are indicated above the bars. Bars of the same graph with different letters are significantly different (*p* < 0.05 by the chi-square test).

**Figure 3:**
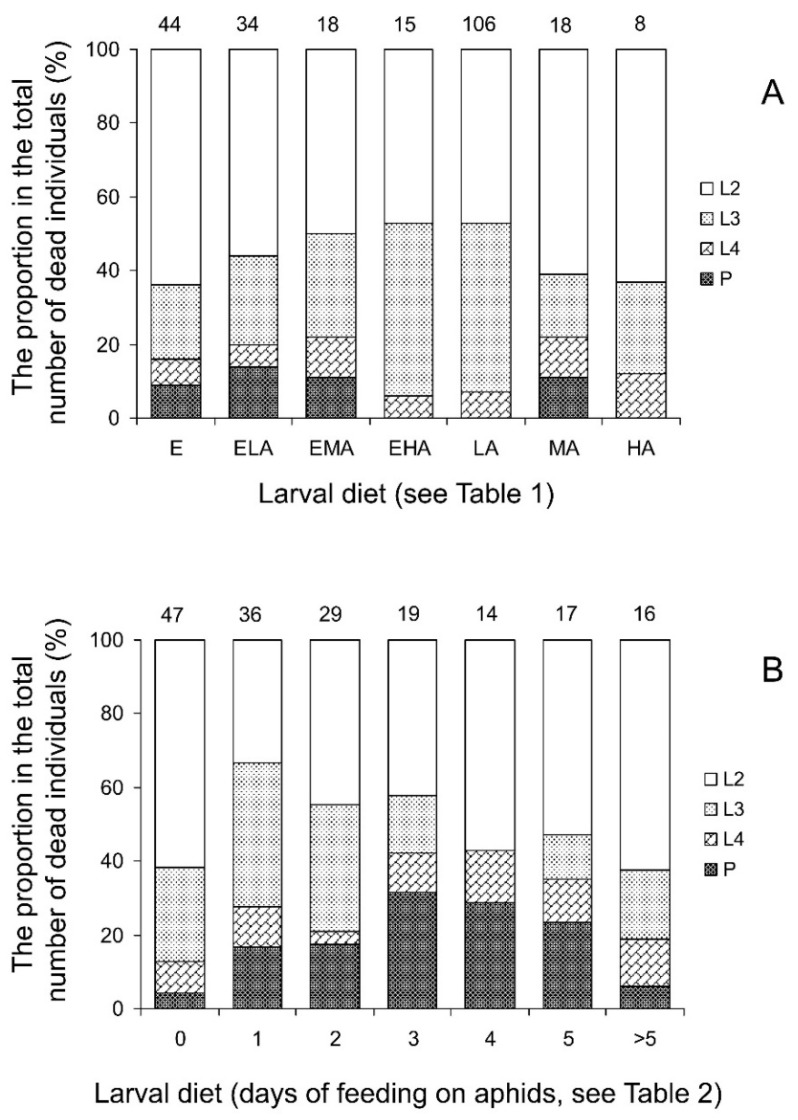
Influence of diet on the structure of *Cheilomenes propinqua* mortality. Diets are indicated below the x-axis. (**A**) The first (mixed diets) experiment; abbreviations of diets: E—eggs of the grain moth, ELA—eggs of the grain moth and low quantity of aphids, EMA—eggs of the grain moth and medium quantity of aphids, EHA—eggs of the grain moth and high quantity of aphids, LA—low quantity of aphids, MA—medium quantity of aphids, HA—high quantity of aphids (see Table 1 for more detailed description of diets). (**B**) The second (changing diets) experiment; the numbers below the x-axis indicate the number of days when the larvae fed on aphids before the switch to feeding on eggs (see Table 2 for more detailed description of diets). Data shown are the percentages of different stages of development in the total number of individuals that died before reaching the adult stage: the second instar larvae (L2), the third instar larvae (L3), the fourth instar larvae (L4), and pupae (P). Sample sizes are indicated above the bars.

A pairwise chi-square test conducted separately for the seven larval diets showed that the influence of the food of maternal females (either aphids or eggs) on the survival of their progeny from the second instar larva to the adult stage was not statistically significant (*p* > 0.05 for all larval diets). Analysis of the pooled data for all diets (chi-square test with the Mantel–Haenszel adjustment, larval diet was used as a strata variable) showed the same result: χ^2^ = 1.48, *df* = 1, *n* = 775, *p* = 0.224. Therefore, the data for survival with the two female diets were pooled for further statistical analysis. The influence of larval diet, in contrast, was highly statistically significant for the total data of the experiment: χ^2^ = 303.9, *df* = 6, *n* = 775, *p* < 0.001. Indeed, as seen in Figure 2A, almost all of the larvae fed on the LA diet died, whereas more than half of the larvae fed on other diets survived to the adult stage. Among them, the highest survival was recorded for the larvae fed on diets with an unlimited supply of aphids (EHA and HA) and the lowest for those fed on grain moth eggs without or with the minimum addition of aphids (E and ELA). Mortality structure, as shown by the chi-square analysis of the total data, was also dependent on the larval diet: χ^2^ = 152.3, *df* = 3, *n* = 243, *p* < 0.001. The general pattern, however, was similar in all treatments of the experiment: the highest mortality occurred at the first and second larval instars (Figure 3A). The influence of maternal diet on the structure of mortality was not statistically significant: χ^2^ = 1.1, *df* = 3, *n* = 243, *p* = 0.776.

The proportion of females in the total progeny was 49.3%, which was not significantly different from the equal distribution: χ^2^ = 0.12, *df* = 1, *n* = 532, *p* = 0.729. Mantel–Haenszel chi-square analysis with the larval diet used as a strata variable showed that the male/female ratio was not dependent on the diet of maternal females: χ^2^ = 0.39, *df* = 1, *n* = 532, *p* = 0.533. Then, the data for the two maternal diets were pooled, and the chi-square analysis of these data showed that the influence of larval diet was also not statistically significant: χ^2^ = 2.24, *df* = 5, *n* = 532, *p* = 0.815.

#### 3.1.2. Time of Development

**Figure 4:**
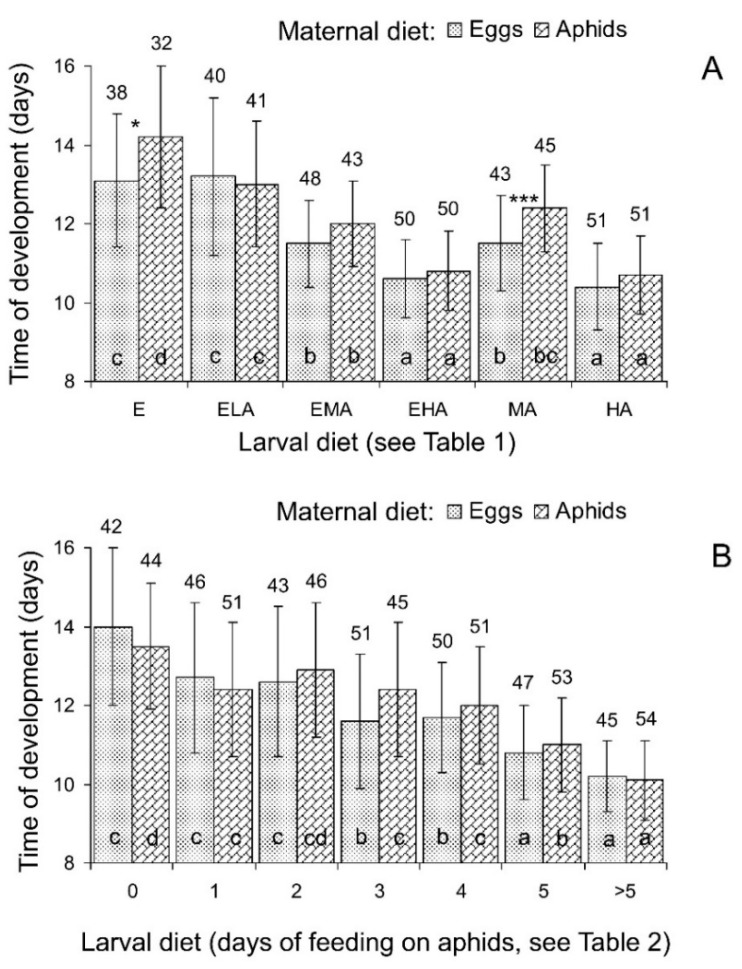
Influence of maternal and larval diets on time of *Cheilomenes propinqua* development from the second instar to the adult stage. (**A**) The first (mixed diets) experiment; abbreviations of diets: E—eggs of the grain moth, ELA—eggs of the grain moth and low quantity of aphids, EMA—eggs of the grain moth and medium quantity of aphids, EHA—eggs of the grain moth and high quantity of aphids, LA—low quantity of aphids, MA—medium quantity of aphids, HA—high quantity of aphids (see Table 1 for more detailed description of diets). (**B**) The second (changing diets) experiment; the numbers below the x-axis indicate the number of days when larvae fed on aphids before the switch to feeding on eggs (see Table 2 for a more detailed description of the diets). Maternal diets are indicated above the graph, larval diets below the x-axis. Data shown are the means and SD. Sample sizes are indicated above the bars. The effect of larval diet: bars of the same graph with the same filling labeled by different letters are significantly different (*p* < 0.05 by Tukey’s HSD test on the ranked data). The effect of maternal diet: asterisks show a significant difference between the neighboring bars (*—*p* < 0.05, ***—*p* < 0.001 by the Kruskal–Wallis non-parametric test).

Three-way ANOVA of the total data (Table 3) showed that the time of *C. propinqua* development from the second larval instar to the adult stage was significantly dependent on both the maternal and larval diets, whereas the influence of sex as well as all interactions were not statistically significant. Hence, the data for males and females were pooled for further statistical analysis. As seen in Figure 4A, the rate of development showed the same pattern of dependence on larval diet as survival (see Figure 2A): the fastest development was recorded for the larvae fed on diets with an unlimited supply of aphids (EHA and HA) and the slowest for those fed on grain moth eggs without or with the minimum addition of aphids (E and ELA), whereas larvae that received the medium daily supply of aphids (EMA and MA) showed intermediate results. With regard to maternal diet, larvae hatched from the eggs laid by females fed on grain moth eggs on average developed somewhat faster than those hatched from the eggs laid by females fed on green peach aphids, although in the pairwise comparisons, this difference was statistically significant for only two larval diets (Table 3 and Figure 4A).

#### 3.1.3. Size and Weight of Emerging Adults

**Figure 5:**
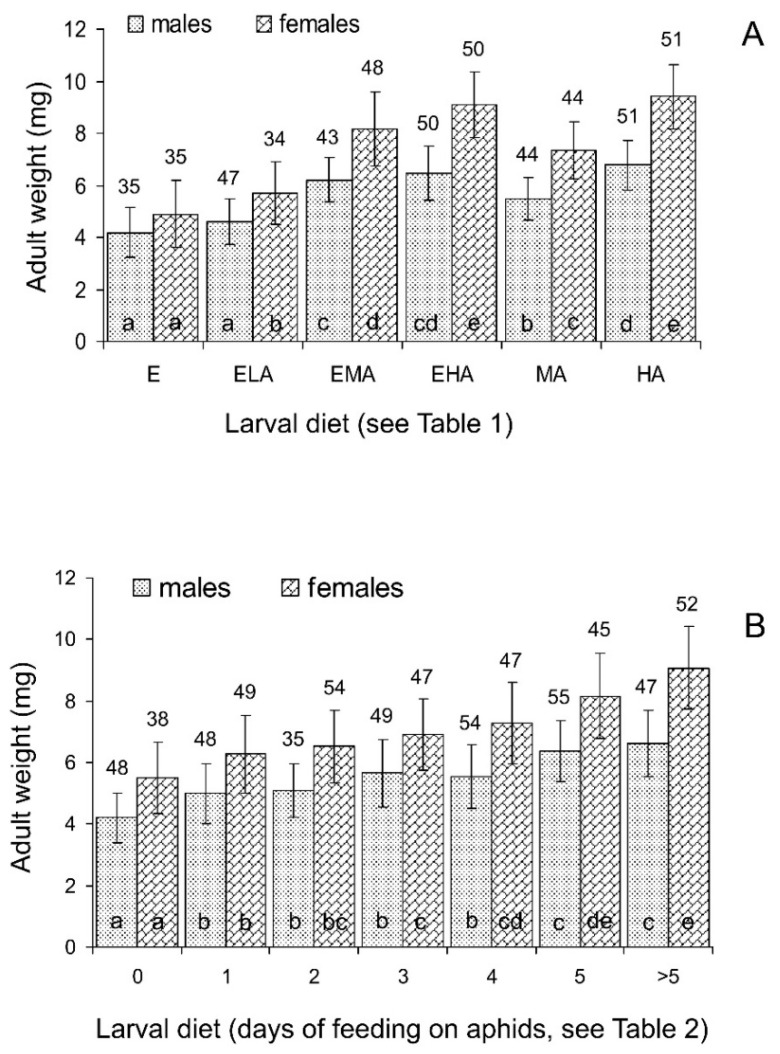
Influence of sex and larval diet on the weight of *Cheilomenes propinqua* emerging adults. (**A**) The first (mixed diets) experiment; abbreviations of diets: E—eggs of the grain moth, ELA—eggs of the grain moth and low quantity of aphids, EMA—eggs of the grain moth and medium quantity of aphids, EHA—eggs of the grain moth and high quantity of aphids, LA—low quantity of aphids, MA—medium quantity of aphids, HA—high quantity of aphids (see Table 1 for more detailed description of diets). (**B**) The second (changing diets) experiment; the numbers below the x-axis indicate the number of days when the larvae fed on aphids before the switch to feeding on eggs (see Table 2 for a more detailed description of diets). Sex is indicated above the graph, larval diets below the x-axis. Data shown are the means and SD. Sample sizes are indicated above the bars. The effect of larval diet: bars of the same graph with the same filling labeled by different letters are significantly different (*p* < 0.05 by the Tukey’s HSD test on ranked data).

**Figure 6:**
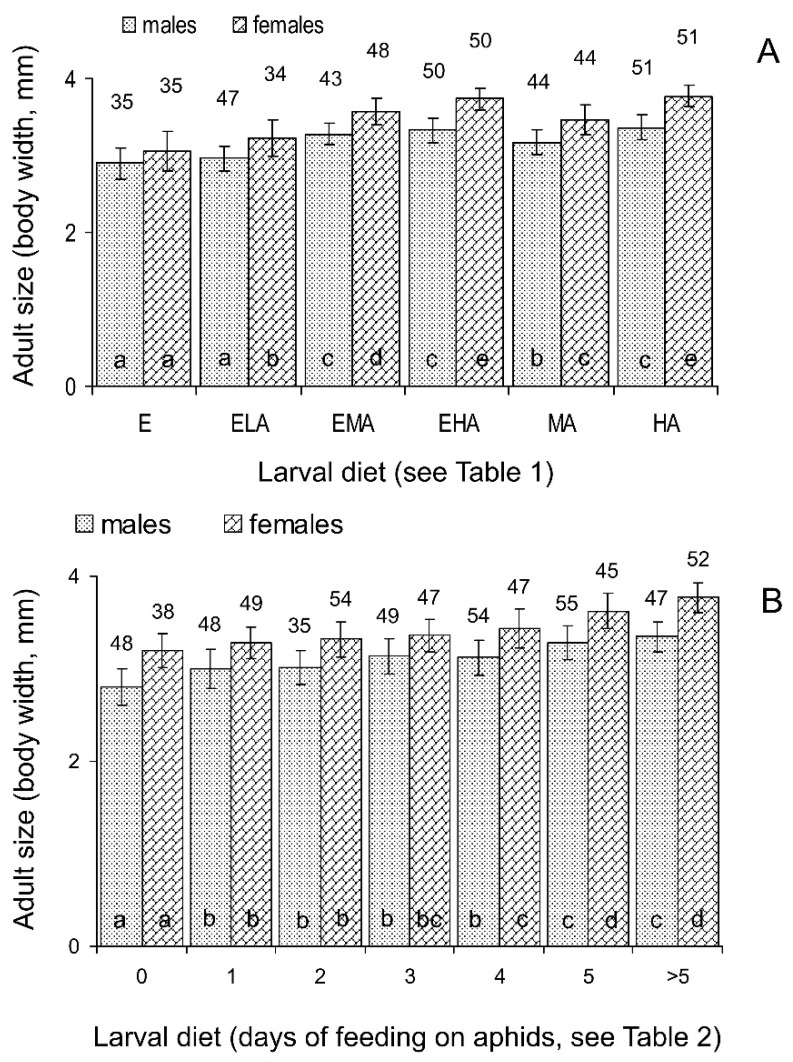
Influence of sex and larval diet on the size of *Cheilomenes propinqua* emerging adults estimated by the maximum body width. (**A**)—the first (mixed diets) experiment; abbreviations of diets: E—eggs of the grain moth, ELA—eggs of the grain moth and low quantity of aphids, EMA—eggs of the grain moth and medium quantity of aphids, EHA—eggs of the grain moth and high quantity of aphids, LA—low quantity of aphids, MA—medium quantity of aphids, HA—high quantity of aphids (see Table 1 for more detailed description of diets). (**B**)—the second (changing diets) experiment; the numbers below the x-axis indicate the number of days when larvae fed on aphids before the switch to feeding on eggs (see Table 2 for more detailed description of diets). Sex is indicated above the graph, larval diets—below the x-axis. Data shown are the means and SD. Sample sizes are indicated above the bars. The effect of larval diet: bars of the same graph with the same filling labeled by different letters are significantly different (*p* < 0.05 by Tukey’s HSD test on the ranked data).

Three-way ANOVA of the total data (Table 3) showed that the weight and size of the emerging *C. propinqua* adults strongly depended on the larval diet and sex of an individual; the interaction of these two factors was also significant, whereas the influence of maternal diet and all other interactions were not statistically significant. Hence, the data on the progeny of females fed on different diets were pooled for further statistical analysis. As seen in Figure 5A and Figure 6A, the size and weight of the males and females showed a similar pattern of dependence on larval diets. Feeding only on eggs (E diet) resulted in the emergence of the smallest and lightest individuals. The addition of the minimum amount of aphids (ELA diet) caused a slight increase in adult size and weight, although this difference was only statistically significant for females. The treatment with the medium amount of aphids (MA diet) yielded somewhat larger and heavier adults, and again, the addition of eggs (EMA diet) resulted in an increase in size and weight (in this case, the difference was statistically significant for both males and females). The largest and heaviest individuals emerged from larvae fed on a high (practically unlimited) amount of aphids (HA diet); the addition of eggs to the unlimited amount of aphids (EHA diet) did not cause any significant changes in adult size and weight (Figure 5A and Figure 6A).

### 3.2. The Second Experiment (Changing Diets)

#### 3.2.1. Pre-Adult Survival and Sex

Analysis of the total data (chi-square test with the Mantel–Haenszel adjustment, larval diet was used as a strata variable) showed that the influence of the food of the maternal females (either aphids or eggs) on the survival of their progeny from the second instar larva to the adult stage was not statistically significant: χ^2^ = 1.09, *df* = 1, *n* = 846, *p* = 0.296. Pairwise chi-square tests conducted separately for the seven larval diets also did not reveal any statistically significant difference (*p* > 0.05 for all cases). Therefore, data for the two female diets were pooled for further statistical analysis. The influence of larval diet, based on the total data of the experiment, was highly statistically significant: χ^2^ = 33.5, *df* = 6, *n* = 846, *p* < 0.001. As seen in Figure 2B, when the number of days of feeding on aphids increased from 0 to 3, the pre-adult survival gradually increased from 64.7 to 83.4% and then remained stable around this level. Further analysis showed that the mortality structure was not significantly dependent on the maternal diet: χ^2^ = 4.1, *df* = 3, *n* = 178, *p* = 0.253. Treatments with different larval diets, as seen in Figure 3B, slightly differed in the structure of mortality, but this difference was not statistically significant: χ^2^ = 25.8, *df* = 18, *n* = 178, *p* = 0.105.

Females constituted 49.7% of the total progeny, which was not significantly different from the equal distribution: χ^2^ = 0.02, *df* = 1, *n* = 668, *p* = 0.877. Mantel–Haenszel chi-square analysis with the larval diet used as a strata variable showed that the proportion of females in the progeny was not dependent on the maternal diet: χ^2^ = 0.47, *df* = 1, *n* = 668, *p* = 0.495. Therefore, the data for the two maternal diets were pooled for chi-square analysis of the total data, which showed that the influence of larval diet was also not statistically significant: χ^2^ = 7.0, *df* = 6, *n* = 668, *p* = 0.322.

#### 3.2.2. Time of Development

Three-way ANOVA of the total data of the second experiment showed that time of *C. propinqua* development from the second larval instar to the adult stage significantly depended on the larval diet, whereas the influence of the diet of maternal females was not statistically significant (Table 4). In addition, males developed somewhat faster than females, but (as demonstrated by statistically insignificant interactions) both sexes showed the same pattern of differences between larval diets. As seen in Figure 4B, the time of pre-adult development decreased with the number of days when the larvae fed on aphids. In contrast to pre-adult survival, a gradual uniform increase in the rate of pre-adult development occurred over the whole range of the used diets (comp. Figure 2B and Figure 4B).

#### 3.2.3. Size and Weight of Emerging Adults

Three-way ANOVA of the total data (Table 4) showed that the weight and size of emerging *C. propinqua* adults strongly depended on the larval diet and sex of an individual; the interaction of larval and maternal diets was marginally significant, whereas the direct influence of maternal diet and all other interactions were not statistically significant. The non-significant interaction of the sex and diet factors suggest that the patterns of weight and size dependence on larval diets in the males and females were similar. This can also be clearly seen in Figure 5B and Figure 6B: both the size and weight of the emerging males and females increased with the number of days of feeding on aphids. Furthermore, for the duration of pre-adult development, this effect was gradual and uniform over the whole range of diets studied.

## 4. Discussion

First, we conclude that the working hypothesis of our study was only partly confirmed. As clearly indicated by the results of the first experiment, feeding on various mixtures of high-quality (green peach aphids) and low-quality (grain moth eggs) foods never resulted in higher pre-adult survival, faster pre-adult development, or a larger size and weight of emerging adults than those observed for unlimited feeding on aphids. The quality of mixed foods was mostly determined by the contents of aphids. In particular, as seen in Figure 2A, Figure 4A, Figure 5A and Figure 6A, the results obtained with feeding on a practically unlimited number of aphids (HA diet) were not significantly different from those with the EHA diet (unlimited number of aphids with an addition of unlimited number of the grain moth eggs). Thus, based on the results of the whole experiment, the additive effect of mixed diets was not detected. However, on the other hand, in some combinations of the limited diets, a significant positive additive effect was shown. For example, when larvae that hatched from eggs laid by females fed on aphids were then fed with the ELA diet (eggs and low quantity of aphids), the time of their development was significantly shorter than those of larvae fed with the E diet (only eggs), whereas larvae fed on the LA diet (only low quantity of aphids) failed to reach the adult stage (Figure 4A). Moreover, the emerged females in the ELA treatments were larger (Figure 5A) and heavier (Figure 6A) than those in the E treatments. Similarly, the males and females that developed from larvae fed with the EMA diet (eggs and medium quantity of aphids) were larger (Figure 5A) and heavier (Figure 6A) than those developed from larvae fed with the E diet (only eggs) and MA diet (medium quantity of aphids). Thus, in some treatments with limited diets, the additive effect of mixed foods was detected.

The experiment with changing diets yielded similar results. The survival, rate of development, adult size, and adult weight gradually increased with the number of days of feeding on aphids. The patterns of these effects differed between the above-mentioned parameters, but the best results were always obtained with the ladybirds constantly fed on aphids, although the difference from the individuals that fed on aphids for 5 days was not always statistically significant.

Some of the earlier studies also failed to demonstrate any significant positive effect produced by mixed, alternating, or changing diets. For example, experiments with larvae of the seven spotted ladybird *Coccinella septempunctata* L. fed on three cereal aphid species (alone or in combination) showed that the survival, rate of pre-adult development, prepupal, and adult weight of individuals fed on a mixed diet were not higher (in some cases even significantly lower) than those for ladybirds fed on the best of pure (unmixed) diets [15]. The same conclusion was later made from a study on the same predator but other aphids: no benefit from the mixing of aphid species was found, and the quality of the mixed diets was determined by the quality of the constituent species [18]. The rate of development of *Hippodamia convergens* (Guerin-Meneville) larvae fed on various mixtures of two aphids (*Schizaphis graminum* (Rondani) and *Rhopalosiphum padi* (L.)) and the weight of the emerged adults were not higher than those of individuals fed on the same quantity of each of the two prey species [19]. Larval survival of two aphidophagous ladybirds, *H. convergens* and *Harmonia axyridis* (Pallas), fed on a mixture of green peach aphids and eggs of the Colorado potato beetle, was significantly lower than when the larvae were fed only with green peach aphids, although in both cases, the aphids were provided in excess [23]. *Brumoides foudrasii* (Mulsant) females fed on high-quality natural food, mealybugs *Ferrisia dasylirii* (Cockerall), had more mature oocytes than females fed on the mixture of *F. dasyliri* and the low-quality factitious food, flower pollen [17]. However, another recent study on *H. convergens* obtained ambiguous results: larvae fed only on *S. graminum* showed faster larval development and a higher weight of emerging adults than larvae fed on the mixture of this prey with plant substances (pollen, sugars, and leaves), but when feeding only on aphids was continued through adult life, the preoviposition period was longer and female fecundity was lower than in individuals fed on the mixed diet [25]. Regarding insect predators from other orders, for instance, the longevity and fecundity of *Orius laevigatus* (Fieber) (Heteroptera, Anthocoridae) females fed on eggs of the grain moth *Sitotroga cerealella* (Oliv.) alternated with cysts of the brine shrimp *Artemia salina* Leach were not higher than those of females fed on the more suitable of the two foods, the grain moth eggs [28].

To justify our unfulfilled expectations, we noted that in many other earlier studies conducted on various species of predatory ladybirds such as *Adalia bipunctata* (L.) [9,10], *Coccinella septempunctata* L. [13], *C. transversalis* (F.) [11], C. *transversoguttata* Brown [13], *Harmonia axyridis* (Pallas) [8,12,20,24], *Heteroneda billardieri* (Crotch) [7], *Hippodamia variegata* (Goeze) [22], *Oenopia conglobata contaminata* (Menetries) [14], and *Propylea japonica* (Thunberg) [16] feeding on mixed or changing diets resulted in higher rates of survival, development, and maturation, larger individuals, and higher fecundity than feeding on each of their components.

Moreover, as noted above, the results of our study cannot be considered as definitely negative. Indeed, the first experiment showed that about 70% of the larvae fed on the ELA diet (the grain moth eggs plus a low quantity of aphids) developed to adults, whereas practically all larvae fed only on a low quantity of aphids (LA diet) failed to reach the adult stage (Figure 1A). Similarly, as seen in Figure 5A and Figure 6A, adults that emerged from the larvae fed on the EMA diet (eggs plus medium quantity of aphids) were significantly heavier and larger than those that emerged from the larvae fed only on a medium quantity of aphids (MA diet). Hence, the possibility of feeding on low-quality food (grain moth eggs) can at least partly compensate for the shortage of high-quality food (aphids). Although this will result in a proportional decrease in pre-adult survival, the rate of development, and the size of emerging adults, these parameters will still be higher than those in the absence of grain moth eggs.

Thus, we conclude for the practice of laboratory and mass rearing that if necessary (for example, in the case of temporary shortage or a lack of aphids), *C. propinqua* larvae can be fed with grain moth eggs by replacing, mixing, or alternating with aphids, although this will inevitably result in a proportional decrease in the pre-adult survival, rate of development, and size of the emerging adults. In the case of preventing release of *C. propinqua* adults for the biological control of pests in greenhouses, larvae hatched from the laid eggs will be able to feed on both aphids or other high-quality prey and supplemented factitious food (the grain moth eggs), but the shortage of high-quality food will lead to the above-mentioned negative consequences. On the other hand, even a minimal addition of aphids can have a substantial positive effect on the development of larvae fed with grain moth eggs. Regarding basic entomology, it is interesting to note that in our study, a significant additive effect was only found in the treatments with a limited supply of high-quality food.

## Figures and Tables

**Table 1:** Composition of diets used in the first (mixed diets) experiment (daily rations are given).

Time of the Experiment ^1^	Diets (Full Names and Abbreviations Used in the Present Paper)						
	Eggs (E)	Eggs and Low Quantity of Aphids (ELA)	Eggs and Medium Quantity of Aphids (EMA)	Eggs and High Quantity of Aphids (EHA)	Low Quantity of Aphids (LA)	Medium Quantity of Aphids (MA)	High Quantity of Aphids (HA)
1st day	Eggs in excess	Eggs + 1 aphid larva	Eggs + 2 adult aphids	Eggs + aphids in excess	1 aphid larva	2 adult aphids	Aphids in excess
2nd day	Eggs in excess	Eggs + 1 aphid larva	Eggs + 4 adult aphids	Eggs + aphids in excess	1 aphid larva	4 adult aphids	Aphids in excess
3rd day	Eggs in excess	Eggs + 1 aphid larva	Eggs + 6 adult aphids	Eggs + aphids in excess	1 aphid larva	6 adult aphids	Aphids in excess
4th day	Eggs in excess	Eggs + 1 adult aphid	Eggs + 8 adult aphids	Eggs + aphids in excess	1 adult aphid	8 adult aphids	Aphids in excess
5th day	Eggs in excess	Eggs + 1 adult aphid	Eggs + 10 adult aphids	Eggs + aphids in excess	1 adult aphid	10 adult aphids	Aphids in excess
6th and subsequent days	Eggs in excess	Eggs + 1 adult aphid	Eggs + 14 adult aphids	Eggs + aphids in excess	1 adult aphid	14 adult aphids	Aphids in excess

^1^ Time of the experiment (days) was counted from molt to the second instar.

**Table 2:** Diets used in the second (changing diets) experiment (daily rations are given, the food was always provided in excess).

Time of the Experiment ^1^	Diets (The Number of Days When the Larvae Fed on Aphids)						
	0	1	2	3	4	5	>5
1st day	Eggs	Aphids	Aphids	Aphids	Aphids	Aphids	Aphids
2nd day	Eggs	Eggs	Aphids	Aphids	Aphids	Aphids	Aphids
3rd day	Eggs	Eggs	Eggs	Aphids	Aphids	Aphids	Aphids
4th day	Eggs	Eggs	Eggs	Eggs	Aphids	Aphids	Aphids
5th day	Eggs	Eggs	Eggs	Eggs	Eggs	Aphids	Aphids
6th and subsequent days	Eggs	Eggs	Eggs	Eggs	Eggs	Eggs	Aphids

^1^ Time of the experiment (days) was counted from molt to the second instar.

**Table 3:** Influence of the maternal female diet, larval diet, and sex of an individual on various biological parameters of *Cheilomenes propinqua* (the results of the first experiment: ANOVA test of the ranked data, *n* = 532, *df*_err_ = 508).

Factor or Interaction of Factors and the Number of Degrees of Freedom	Biological Parameters		
	Time of Development from the Second Instar to the Adult Stage	Adult Size	Adult Weight
Female diet, *df* = 1	*F* = 17.5, *p* < 0.001	*F* = 2.3, *p* = 0.134	*F* = 1.5, *p* = 0.217
Larval diet, *df* = 5	*F* = 68.7, *p* < 0.001	*F* = 152.8, *p* < 0.001	*F* = 151.7, *p* < 0.001
Sex, *df* = 1	*F* = 2.7, *p* = 0.098	*F* = 433.1, *p* < 0.001	*F* = 357.7, *p* < 0.001
Female diet × larval diet, *df* = 5	*F* = 1.8, *p* = 0.101	*F* = 0.8, *p* = 0.576	*F* = 0.4, *p* = 0.851
Female diet × sex, *df* = 1	*F* = 0.7, *p* = 0.415	*F* = 0.0, *p* = 0.841	*F* = 0.7, *p* = 0.395
Larval diet × sex, *df* = 5	*F* = 0.9, *p* = 0.503	*F* = 7.3, *p* < 0.001	*F* = 7.8, *p* < 0.001
Female diet × larval diet × sex, *df* = 5	*F* = 0.6, *p* = 0.722	*F* = 1.3, *p* = 0.255	*F* = 1.6, *p* = 0.153

**Table 4:** Influence of the maternal female diet, larval diet, and sex of an individual on various biological parameters of *Cheilomenes propinqua* (the results of the second experiment: ANOVA test of the ranked data, *n* = 668, *df*_err_ = 640).

Factor or Interaction of Factors and the Number of Degrees of Freedom	Biological Parameters		
	Time of Development from the Second Instar to the Adult Stage	Adult Size	Adult Weight
Female diet, *df* = 1	*F* = 1.74, *p* = 0.187	*F* = 0.7, *p* = 0.437	*F* = 0.3, *p* = 0.580
Larval diet, *df* = 6	*F* = 58.2, *p* < 0.001	*F* = 85.9, *p* < 0.001	*F* = 75.8, *p* < 0.001
Sex, *df* = 1	*F* = 7.3, *p* = 0.007	*F* = 426.3, *p* < 0.001	*F* = 323.6, *p* < 0.001
Female diet × larval diet, *df* = 6	*F* = 1.1, *p* = 0.346	*F* = 2.3, *p* = 0.036	*F* = 2.2, *p* = 0.042
Female diet × sex, *df* = 1	*F* = 0.1, *p* = 0.893	*F* = 0.7, *p* = 0.419	*F* = 0.6, *p* = 0.450
Larval diet × sex, *df* = 6	*F* = 1.3, *p* = 0.264	*F* = 1.0, *p* = 0.432	*F* = 0.9, *p* = 0.461
Female diet × larval diet × sex, *df* = 6	*F* = 0.8, *p* = 0.523	*F* = 0.6, *p* = 0.760	*F* = 0.4, *p* = 0.833

## Data Availability

The data presented in this study are available on request from the corresponding author.
